# Chloroquine and Chemotherapeutic Compounds in Experimental Cancer Treatment

**DOI:** 10.3390/ijms25020945

**Published:** 2024-01-12

**Authors:** Natalia I. Agalakova

**Affiliations:** Sechenov Institute of Evolutionary Physiology and Biochemistry, Russian Academy of Sciences, 44 Thorez Avenue, Saint-Petersburg 194223, Russia; nagalak@mail.ru

**Keywords:** chloroquine, hydroxychloroquine, autophagy, chemotherapy, cultured cancer cells, animal xenografts, clinical trials

## Abstract

Chloroquine (CQ) and its derivate hydroxychloroquine (HCQ), the compounds with recognized ability to suppress autophagy, have been tested in experimental works and in clinical trials as adjuvant therapy for the treatment of tumors of different origin to increase the efficacy of cytotoxic agents. Such a strategy can be effective in overcoming the resistance of cancer cells to standard chemotherapy or anti-angiogenic therapy. This review presents the results of the combined application of CQ/HCQ with conventional chemotherapy drugs (doxorubicin, paclitaxel, platinum-based compounds, gemcitabine, tyrosine kinases and PI3K/Akt/mTOR inhibitors, and other agents) for the treatment of different malignancies obtained in experiments on cultured cancer cells, animal xenografts models, and in a few clinical trials. The effects of such an approach on the viability of cancer cells or tumor growth, as well as autophagy-dependent and -independent molecular mechanisms underlying cellular responses of cancer cells to CQ/HCQ, are summarized. Although the majority of experimental in vitro and in vivo studies have shown that CQ/HCQ can effectively sensitize cancer cells to cytotoxic agents and increase the potential of chemotherapy, the results of clinical trials are often inconsistent. Nevertheless, the pharmacological suppression of autophagy remains a promising tool for increasing the efficacy of standard chemotherapy, and the development of more specific inhibitors is required.

## 1. Introduction

**Figure 1:**
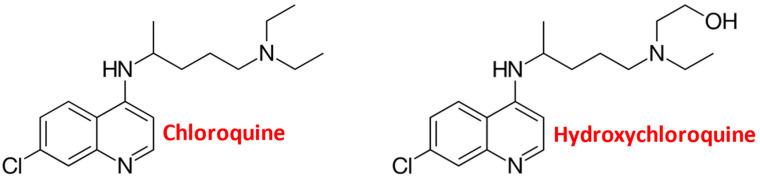
Chemical structure of chloroquine and hydroxychloroquine.

Chloroquine (CQ) and its derivative hydroxychloroquine (HCQ) are synthetic analogs of a world-famous medicinal herb extract, quinine, with a few centuries of antimalarial history [1,2,3]. They belong to a group of 4-aminoquinoline derivatives and possess the property of amphiphilic weak bases. HCQ differs from CQ by one hydroxyl group, the addition of which results in decreased toxicity with the same efficacy (Figure 1). CQ was synthesized in 1934 by Hans Andersag and initially introduced in clinical practice in 1947 due to its significant therapeutic value as an antimalarial agent. Since then, it has been widely used as the first-line medicine for prophylactics and the treatment of uncomplicated malaria caused by a few susceptible strains of *Plasmodium* parasites. However, during the last few decades, CQ and HCQ have been probed for a variety of other diseases. These drugs have a wide therapeutic index and well-established dose safety profiles, and they are inexpensive and orally bioavailable, thus attracting the substantial interest of researchers and clinicians [4]. CQ was shown to be effective for anti-intestinal amebiasis caused by trophozoites of *Entamoeba histolytica*, which causes amebic dysentery [5]. Both CQ and HCQ have been successfully used for the treatment of autoimmune diseases like rheumatic diseases [2,6] and systemic lupus erythematosus [6,7,8]. Recently, they have also been tested for the treatment and prophylactics of viral infections, including Zika virus [9,10], human immunodeficiency virus (HIV) [11,12], and COVID-19, although the obtained results were inconsistent or negligible and revealed many side effects [13,14].

Most importantly, CQ and HCQ have been intensively investigated as potential tools for the treatment of cancers of various origins [3,4,15,16]. Antitumor CQ/HCQ activity as a single agent or as adjuvant therapy in combination with widely used cytotoxic compounds has been probed in a long list of malignancies. This review focuses on the findings of a series of experimental in vitro and in vivo studies that tested CQ or HCQ as additives to conventional chemotherapy. A few examples of results obtained in completed clinical trials published in scientific journals and providing detailed information about the number of patients and types of tumors are included as well. For a more comprehensive review of both completed and ongoing clinical trials that have applied CQ and HCQ for the treatment of various cancers, the readers are referred to other recent works [17,18,19]. The effects of CQ/HCQ on cultured cancer cells, on various animal tumor xenografts, and on tumors in clinical practice are summarized in the tables. In experimental settings, CQ application outnumbered HCQ, while the majority of clinical trials used HCQ for combination therapy due to its lower toxicity.

## 2. Cellular Chloroquine Effects

The major molecular mechanism believed to underly antitumor CQ and HCQ effects and make them potential tools for cancer therapy is their ability to suppress autophagy [3,15,16]. Autophagy is an evolutionarily conserved intracellular process necessary for the maintenance of cellular homeostasis and the selective recycling of damaged proteins, macromolecular complexes, or whole organelles into lysosomes. Under conditions of nutrient deprivation or stress, autophagy is stimulated to supply the cells with an alternative energy source, thus promoting temporary survival [20,21]. A key process of autophagy is a transient generation of phagophores, sequestering structures that engulf unwanted cellular material and mature into double-membrane autophagosomes. Further fusion with lysosomes allows cargo degradation and turnover. The major molecular players of autophagy are Beclin-1, p62/SQSTM1 degrading scaffold protein, marker of autophagosomes LC3-II, and ATG proteins, which phosphorylate autophagy-related effectors and form the phagophores and autophagosomes.

Autophagy was implicated in the progression of cancers of different origins, with it at higher levels closely correlating with lower overall survival. However, its roles in these malignancies are complicated, as it can work as either a promoter or suppressor of cell death depending on the stage and type of cancer [22,23,24]. By recycling the accumulated metabolites and positively regulating the metabolism of cancer cells, autophagy can function as a self-protective response against antitumor compounds, thus being a critical factor in the development of resistance to chemotherapy. On the other hand, recent studies indicate that a series of mutations such as RAS, BRAF, and p53 can alter the vulnerability of cancer cells to death and their sensitivity to cytotoxic drugs. Thus, chemotherapy-induced autophagy emerges as a promising critical target. It is believed that its suppression leads to the accumulation of autophagosomes, which can compromise cell viability and trigger apoptosis.

**Figure 2:**
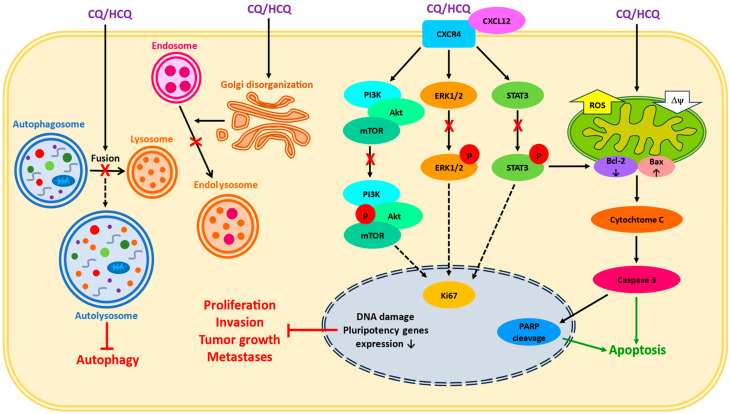
A simplified scheme of reported CQ/HCQ effects in cancer cells. The impact on lysosomal and endosomal systems, the disturbances in intracellular signaling, and the induction of mitochondria-dependent apoptosis are presented. X—inhibition, ROS—reactive oxygen species, Δψ—mitochondrial membrane potential, p—phosphorylation.

CQ and HCQ are lysosomotropic agents, which suppress the final step of autophagy by inhibiting the fusion of late endosomes with lysosomes (Figure 2). After entering the cells, they passively diffuse into subcellular structures responsible for protein synthesis and recycling—Golgi vesicles, endosomes, and lysosomes. In acidic environments, they undergo protonation and remain trapped inside, thus causing alkalinization. This process inhibits the ability of enzymes to degrade unwanted material and blocks the survival mechanisms in cancer cells which allows them to proliferate [3,4].

However, CQ/HCQ are not specific autophagy inhibitors, as they can affect other cellular processes beyond autophagy (Figure 2). Among their reported therapeutic effects on cancer cells are autophagy-independent disturbances in chemokine signaling, increased ROS production, mitochondria damage, the induction of apoptosis, modifications in the tumor microenvironment, the normalization of tumor-associated vascularization, the prevention of pro-thrombotic processes, the activation of antitumor immune responses, the inhibition of tumor-promoting intermediates via tumor-associated macrophages, the negative modulation of cancer-associated fibroblasts, the modulation of metabolic responses, the alteration of intracellular calcium balance, and the disruption of membrane stability [2,3,4].

## 3. Chloroquine as a Single Treatment

In many in vitro (Table 1) and in vivo studies, the application of CQ or HCQ as single agents has been found to effectively activate the cellular antitumor mechanisms, leading to both the induction of apoptosis and the suppression of autophagy. CQ inhibited the growth of orthotopic U87MG glioblastoma in a mouse model, whereas the decreased viability of cultured glioma cells was accompanied by the stimulation of caspase-3, pro-apoptotic protein Bax, and the p53 death pathway [25]. Lakhter et al. [26] showed that CQ reduces the growth of melanoma SKMe123 cells and mice melanoma xenografts via the lysosome-independent induction of apoptosis and prevention of PUMA protein degradation. The diminished tumorigenicity of primary pancreatic duct adenocarcinoma cells (PDAC) in the presence of CQ was a result of the inhibition of chemokine receptors CXCL12/CXCR4 and hedgehog signaling pathways accompanied by the downregulation of pluripotency-related genes. Such events led to the depletion of the cancer stem cells (CSCs) pool, although CQ had no effect on the growth of primary patient-derived pancreatic cancer xenografts in vivo [27]. Moreover, CQ did not increase the LC3-II level in primary PDAC but inhibited autophagy in Panc1, 8988 T, and BxPC3 cell lines [27]. The in vitro CQ treatment of liver HepG2 cancer cells resulted in G0/G1 cell cycle arrest, DNA damage, the activation of caspase-3 and pro-apoptotic protein Bim, PARP cleavage, and the loss of mitochondrial membrane potential, while an injection of CQ to mice bearing HepG2-GFP human liver cancer cells suppressed tumor growth [28]. An addition of CQ to a pancreatic neuroendocrine neoplasm (PanNEN) culture induced ER stress and unfolded protein response via the activation of the PERK-eIF2α-ATF4 pathway, resulting in the expression of pro-apoptotic protein CHOP. In Men1 heterozygous-deficient (Men1^+/ΔN3-8^) mice, a mouse PanNEN model, HCQ administration decreased tumor size and accelerated apoptosis, although proliferative activity was unchanged [29]. In patient-derived glioblastoma stem cell lines with or without p53 mutations, CQ-suppressed proliferation was accompanied by the decreased activity of ATM (ataxia-telangiectasia mutated) and HIPK2 (homeodomain-interacting protein kinase*)* kinases functioning as modulators of p53-mediated transcription [30]. However, the survival of mice bearing glioblastoma xenografts following CQ administration greatly depended on p53 mutations [30]. In human cervical cancer HeLa cells and osteosarcoma U2OS cells, CQ treatment induced the autophagy-independent disorganization of Golgi systems [31]. The compromised mammosphere-forming efficiency of triple-negative breast cancer (TNBC) Hs578t, MDAMB231, and SUM159PT cells exposed to CQ in vitro and anti-metastasizing CQ effects in a mouse TNBC xenograft model were associated with a reduction in the tumorigenic CD44^+^/CD24^−/low^ stem cell population accompanied by the inhibition of Jak2 and STAT3 phosphorylation, global DNA hypomethylation and damage, oxidative stress, mitochondrial membrane depolarization, and the release of cytochrome C to cytosol [32,33]. In a few cultured cell lines of adult T-cell leukemia/lymphoma (ATT) and a mouse Su9T01 tumor xenograft model, CQ or HCQ exerted a pronounced antitumor effect by rescuing the p47 protein, a negative regulator of the NF-κB pathway, from autophagy-lysosomal degradation, and via the downregulation of CADM1 (cell adhesion molecule 1) [34].

The direct effect of CQ/HCQ on autophagy was confirmed in a series of other works. Thus, an increased number of autophagosomes and late endosomes, as well as an upregulation of LAMP, p62, and LC3-II proteins, have been reported in HeLa [31], U2OS [31], and TNBC cells [32,33]. The compromised proliferation and colony formation of endometrial adenocarcinoma cells with or without p53 mutations and an increased population of apoptotic cells after CQ treatment were also accompanied by the accumulation of autophagosomes, endosomes, LC3, and p62 [35]. In human bladder cancer cell lines (RT4, 5637, and T24), CQ or HCQ inhibited proliferation and clonogenic formation via DNA fragmentation, increased apoptosis, the stimulation of caspases 3/7, PARP cleavage, the suppression of lysosome fusion, the accumulation of p62 and LC3-II [36]. A similar inhibition of autophagy and stimulation of apoptosis was shown in brain [30,37], ovarian [38], breast [39,40], thyroid [41], and ATT [34] cancer cells.

**Table 1:** The effects of single CQ treatment on cultured cancer cells of different origins.

Agent	Experimental System	Treatment Regime	Effects	Molecular Markers	Reference
CQ	Glioma U87MG, U251, G120, G130, and G44 cells	10–40 µg/mL for 24–72 h	↓Cell growth ↓Viability	↑Caspase 3 ↑p53 ↑Bax	[25]
CQ	Melanoma SK-MEL23 and VMM39 cells	25–50 µM for 5–28 h	↓Viability, ↓Lysosomal activity ↓Autophagy ↑Apoptosis	↑Caspase 3 ↑PUMA ↑p62 ↑LC3	[26]
CQ	Primary pancreatic cancer cells	10 µM for 7 days	↓CSCs number ↓Sphere-forming ability ↓CSCs pool in spheres ↓Invasiveness	↓CXCL12/CXCR4 signaling ↓Hedgehog signaling ↓p-ERK and p-STAT3 ↓Expression of pluripotency- related genes *OCT4*, *SOX2*, *NANOG*, and *cyclins D1* and *E1*	[27]
CQ	HepG2 and Huh7 human liver cancer cells	10–30 µM for 24–72 h	↓Proliferation, ↑Apoptosis G0/G1 cell cycle arrest	DNA damage ↑Caspase-3, cleaved PARP, and Bim ↓Mitochondrial membrane potential	[28]
CQ	Pancreatic neuroendocrine neoplasm		↑ER stress ↑Apoptosis	↑PERK, eIF2α, ATF4, and CHOP	[29]
CQ	Patient-derived glioblastoma stem cell lines no. 993, G112SP and no. 1095	CQ 30 µM for 24–72 h	↓Proliferation ↓Viability	↓Ki67 ↑SubG1 fraction ↑p53, p21, and caspase-3 ↓HIPK2 and ATM ↓p-Akt ↑LC3-II and p62	[30]
CQ	Human cervical cancer HeLa cells	100 µM for 2–5 h	↓Autophagy	↑Autophagosomes Disorganization of Golgi and endo-lysosomal systems	[31]
CQ	Osteosarcoma U2OS cells	100 µM for 2–5 h	↓Autophagy	Disorganization of Golgi and endo-lysosomal systems ↑LC3-II, p62/SQSTM1, and LAMP	[31]
CQ	Triple-negative breast cancer Hs578t, MDAMB231, and SUM159PT cells	1 µM for 48 h	↓Mammosphere-forming efficiency ↓CD44^+^/CD24^−/low^ stem cells population ↓Autophagy ↓DNA methylation	↑Autophagosomes ↑LC3, p62, and caspase-3 ↓STAT3 and Jak2 phosphorylation ↓DNMT1	[32]
CQ	Triple-negative breast cancer Hs578t, MDAMB231, and SUM159 cells	10–20 μM for 48 h	↓Autophagy ↓CD44^+^/CD24^−/low^ CSCs number Mitochondrial damage Cristae vacuolization DNA damage	Mitochondrial membrane depolarization Cytochrome C release ↑LC3 and p62 ↑Superoxide ↓Cytochrome C oxidase and NQO1 ↑γ-H2AX	[33]
CQ HCQ	Adult T-cell leukemia/lymphoma (ATLL) cell lines	CQ 50 µM or HCQ 25 µM for 6–24 h	↓Viability and growth ↓Autophagy ↑Apoptosis	↑Caspase-3, LC3 ↑Autophagosomes ↑p47 and IκBα ↓NEMO, CADM1	[34]
CQ	Endometrial cancer AN3CA, KLE, and Ishikawa cells	0.5–20 µM for 24–72 h	↓Proliferation ↓Colony formation ↓Autophagy ↑Apoptosis Cell cycle arrest	↑Cleaved caspase-3 ↑LC3-I, LC3-II, and p62 ↑Autophagosomes and endosomes	[35]
CQ, HCQ	Bladder cancer RT4, 5637, and T24 cells	CQ 25 µM or HCQ 20 µM for 24–72 h	↓Viability ↓Clonogenic ability ↓Autophagy ↑Apoptosis	↑Caspase3/7 activity; ↑Cleaved PARP ↑LC3-II and p62 ↓Lysosome fusion DNA fragmentation	[36]
CQ	Vemurafenib-resistant brain tumor 794R and AM38R cells	CQ 5 or 10 μM for 6 or 96 h		↑LC3-II	[37]
CQ	Epithelial ovarian CSCs	10–50 µM for 72 h or 2–10 µM for week	↓Viability ↓Adhesion ↓Spheroid cell viability and diameter		[38]
CQ	Breast cancer MCF-7 cells	16–256 µM for 48 h	↓Viability and growth		[39]
CQ	Breast cancer MCF-7 cells	32.5 µM for 48 h	↓Viability and growth ↑Apoptosis ↓Autophagy	DNA damage Cytochrome C release ↑Autophagosomes ↑Bax, p53 ↑Caspases 3 and 9 mRNA	[40]
CQ	Thyroid cancer TPC1, ATC1, and KTC1 cells	50 µM for 48 h	↓Viability ↓Autophagy ↑Apoptosis	↑LC3 and p62 DNA damage	[41]

Comments: ↑—increased expression, ↓—downregulation.

## 4. Chloroquine and Chemotherapy Drugs

### 4.1. Chloroquine and Doxorubicin (DOX)

Doxorubicin (DOX), a member of the Anthracyclines family, is widely used in chemotherapy against a variety of malignancies such as breast, genitourinary, and ovarian cancers; Hodgkin’s and non-Hodgkin’s lymphomas; Ewing and soft tissue sarcoma; lymphocytic and myelogenous leukemias; gastrointestinal, liver, and thyroid cancers; and neuroblastoma [42,43]. The molecular mechanisms of DOX's impact on cancer cells include intercalation into the DNA–topoisomerase II complex, which causes DNA damage, followed by p53-mediated cell cycle arrest, alterations in the redox state due to ROS accumulation and iron-dependent lipid peroxidation, the dysregulation of calcium-binding proteins and channels, and increased production of interleukins and interferons facilitating the immune-driven clearance of tumor cells. However, severe DOX cardiotoxicity leading to the death of cardiomyocytes and endothelial cells via autophagy, ferroptosis, necroptosis, or pyroptosis limits the benefits of DOX therapy [44]. Moreover, long-term DOX therapy was reported to be associated with the development of resistance due to the activation of autophagy [45,46].

Combined applications of CQ or HCQ with DOX in in vitro and in vivo studies have confirmed the effectiveness of autophagy suppression in overcoming DOX resistance (Table 2 and Table 3). In human hepatocellular carcinoma cells, an addition of a non-toxic CQ dose potentiated DOX cytotoxicity by diminishing its IC50 and preventing DOX-induced autophagy, evident from an increased LC3-II/LC3-I ratio and p62 expression [47]. Co-treatment with CQ significantly sensitizes melanoma cells to DOX in vitro via the suppression of autophagy and enhancement of pyroptosis accompanied by the generation of the plasma membrane-targeting DFNA5-N fragment of gasdermin family protein DFNA5 [48]. In cultured MCF-7 human breast cancer cells and the MCF-7 xenograft mouse model, CQ increased the sensitivity to DOX treatment and suppressed cell growth and aggressiveness via the downregulation of the Ki67 protein, a nuclear marker of active proliferation, the PPT1 enzyme involved in lysosomal degradation, and PI3K/Akt/mTOR signaling pathways [39,40,49]. In TNBC HCC1806 cells, however, although DOX/CQ co-treatment reduced DOX doses and potentiated the growth inhibitory effect, such exposure also inhibited apoptosis, indicating the existence of alternative death pathways [50]. Bano et al. [51] showed an ability of CQ to enhance anticancer DOX effects in cervical cancer HeLa cells, where the synergistic effect was associated with the cleavage of procaspase-3 and PARP, upregulation of p62 and LC-3II, and decreased expression of LAMP-2, Syntaxin17, Rab5, and Rab7 proteins, which play a critical role in the fusion of autophagosomes with lysosomes. In human adenocarcinoma alveolar basal A549 cells, CQ accelerated DOX-induced apoptosis mediated by oxidative stress and led to the dephosphorylation of ERK kinases [52]. DOX/CQ administered to mice inoculated with Ehrlich ascites carcinoma cells partially prevented the disruption of the alveolar structure, reduced the levels of antioxidant enzymes, and increased the level of neutrophil gelatinase-associated lipocalin (NGAL) playing an important role in bacterial defense and inflammation [53]. Moreover, CQ therapy enhanced the anti-angiogenic effect of DOX in HUVECs [54]. However, in thyroid cancer cell lines (TPC1, ACT1, and KTC1), CQ failed to enhance the efficacy of DOX [41].

DOX/CQ was also tested in a series of new formulations proposed to decrease their doses and overcome prominent hydrophobicity [3,55,56]. One such compound is PEGylated (poly(ethylene glycol)-coated) liposomal DOX (PLD) with a prolonged circulation time and increased microvascular permeability but without apparent cardiac toxicity [42,57]. A combination of CQ with PLD and pulse-wave ultrasound hyperthermia (pUH), a scheme developed to enhance the delivery of drugs to subcutaneous 4T1 breast cancer explant in BALB/c mice, induced the long-term suppression of tumor growth in comparison to CQ monotherapy or PLD + pUH treatment [58,59]. In HeLa cells, CQ enhanced the cytotoxicity of DOX encapsulated in pH-sensitive liposomes (SpHL-DOX) created to accelerate drug delivery in acidic environments [60]. DOX/CQ co-loading in polyglycerol functionalized MoS_2_ nanosheets (DOX/CQ-FPMoS_2_) designed for targeted delivery and chemo-photothermal therapy enhanced the anticancer effect of laser irradiation in multidrug-resistant HeLa (HeLa-R) cells [61]. The delivery of simultaneously encapsulated DOX⋅HCl and CQ in pH-responsive cholesteryl hemisuccinate self-assembled nanovesicles (DC-DIV/C) to DOX-resistant K562/ADR, and MCF-7/ADR cells or nude mice bearing a drug-resistant K562/ADR xenograft led to a much stronger antitumor effect accompanied by apoptosis and the blockage of autophagosome and lysosome fusion [62].

**Table 2:** The effects of CQ in combination with chemotherapy drugs on cultured cancer cells of different origins.

Agent	Experimental System	Treatment Regime	Effects	Molecular Markers	Reference
CQ + DOX	Breast cancer MCF-7 cells	DOX 0.05–0.2 µM + CQ 16–64 µM for 48 h	↑Sensitivity to DOX ↓Viability and growth		[39]
CQ + DOX	Breast cancer MCF-7 cells	DOX 3.38 µM + CQ 32.5 µM for 48 h	↑Sensitivity to DOX ↓Viability and growth ↑Apoptosis ↓Autophagy	DNA damage Cytochrome C release ↑Autophagosomes ↑Bax, p53, and caspases 3 and 9 ↑Beclin-1, ATG7, LC3-II, and p62 ↓PI3K, Akt, mTOR, and Bcl-2	[40]
CQ + DOX	Hepatocellular cancer HepG2, Huh7, SNU387, and SNU449 cells	DOX 0.25–1 μg/mL + CQ 20 μM for 48 h	↑DOX cytotoxicity ↓Viability ↓Autophagy	↑LC3 and p62	[47]
CQ + DOX	Melanoma SK-MEL-5, SK-MEL-28, and A-375 cells	DOX 1–2.5 μM + CQ 20 μM for 24 h	↑Pyroptosis ↓Autophagy ↓Viability	↑Cleaved caspase-3 ↑N-DFNA5	[48]
CQ + DOX	Breast cancer MCF-7 cells	DOX 0.17 µM + CQ 16–256 µM for 48 h	↓Viability and proliferation	↓Viability ↓PPT1 expression	[49]
CQ + DOX	Cervical cancer HeLa cells	DOX 40 nM + CQ 40 µM	↑Sensitivity to DOX ↑Apoptosis ↓Autophagy	↑p62, LC3-II, caspase-3, and PARP ↓LAMP-2, Syntaxin 17, Rab 5, and Rab 7	[51]
CQ + DOX	Human umbilical vein endothelial cells (HUVECs)	DOX 01–1 µM + CQ 0.25–32 µM for 48 h	↑Anti-angiogenic effect of DOX		[54]
CQ + SpHL-DOX	Cervical cancer HeLa cells	SpHDL-DOX 3.22 µM + CQ 20 μM for 4 h	↓Viability ↑Apoptosis		[60]
CQ + DOX@ FP-MoS_2_	Cervical cancer HeLa-R cells	DOX 5 μg/mL + CQ 5 μg/mL + FP-MoS_2_ 40 μg/mL for 48 h	↓Viability ↑Transfer and accumulation in tumor cells		[61]
CQ + DOX HCl in DC-DIV/C	DOX-resistant MCF-7/ADR and K562/ADR cells	DOX 5 μg/mL + CQ 10 μg/mL for 24–48 h	↑Sensitivity to DOX ↑Apoptosis ↓Autophagy	↑Autophagosomes ↑LC3-II and p62	[62]
CQ + PTX	TNBC Hs578t, MDAMB231, and SUM159PT cells	PTX 5 nM + CQ 1 µM for 48 h	↑Sensitivity to PTX ↓Autophagy ↓CD44^+^/CD24^−/low^ stem cells population ↓Sphere-forming capacity ↓DNA methylation	↑Autophagosomes ↑Cleaved caspase-3 ↑LC-3II and p62 ↓p-STAT3 and p-Jak2 ↑SOCS1 and SOCS3 ↓DNMT1	[32]
CQ + PTX	Breast cancer MCF-7 cells	PTX 1.5–3 nM + CQ 32–64 µM for 48 h	↓Viability and growth		[39]
CQ + CIS	CIS-resistant endometrial cancer Ishikawa cells	CIS 0.01–100 µM + CQ 1 µM for 72 h	↑Sensitivity to CIS		[35]
CQ + CIS	Thyroid TPC1, ACT1, and KTC1 cells	CIS 2 µM + CQ 50 µM for 48 h	↑Apoptosis ↓Autophagy	↑LC3 and p62	[41]
CQ + CIS	Human neuroblastoma SH-SY5Y	CIS 2 µM + CQ 15 µM for 48 h	↑Apoptosis ↑CIS sensitivity	↑LC3-II/LC3-I and p62	[63]
CQ + CIS	Epithelial ovarian cancer SKOV3 and hey cells	CIS 2.5–10 µM + CQ 5–10 µM for 24–48 h	↓Viability, migration and invasion ↑Apoptosis	↑Autophagosomes ↑Bax and LC3-II/LC3-I ↑Cleaved caspase-3 and PARP ↓Bcl-2 and Bcl-XL	[64]
HCQ + CIS	Human neuroblastoma SH-SY5Y	CIS 0.5–2 µM + HCQ 1 µg/mL for 24–48 h	↑Apoptosis ↓Autophagy	↑LC3-II ↑ROS	[65]
CQ + CPT	TNBC SUM159 SCSs	CPT 10 µM + 10 µM CQ for 48 h	Additive CQ effect ↓CD44^+^/CD24^−/low^ DNA damage	↓Rad50 and Rad51 ↑Cleaved PARP and Bcl-2	[33]
CQ + OXP	Hepatocellular carcinoma HepG2 transfected with *ATG7* shRNA	OXP 18 µM + CQ 80 µM for 12–48 h	↑Apoptosis	↑AVOs ↑LC3 ↑caspase-3	[66]
CQ + OXP	Colon cancer HT29 cells	OXP 0.95–1.6 µM + CQ 1–5 µM for 24 h	↑Sensitivity to OXP ↓Autophagy	↓LC3 staining	[67]
TH-NP with HCQ + OXP	Hepatocellular carcinoma HepG2, Huh-7, and HCCLM3 cells	OXP 20 µM + HCQ 10 µM for 24 h	↓Autophagy ↓Proliferation ↓Colony formation ↓Invasion and migration	↑LC3-I, LC3-II, and p62 ↑E-cadherin, Paxillin, and PARP ↑Autophagosomes	[68]
CQ + GEM	Gallbladder cancer cells GBC-SD, SGC-996, and NOZ	GEM 20 µM + CQ 10 µM for 48 h	↑Antitumor GEM effect ↑Apoptosis ↓Viability ↓Colony formation Cell cycle arrest	↑Bax, LC3-II/LC3-I, and p62 ↓Bcl-2 and PARP ↓p-Akt and p-mTOR	[69]
CQ + GEM	Pancreatic cancer PANC-1 cells	GEM 20 µM + CQ 10 µM for 72 h	↓Viability		[70]
PDGL-GEM@CAP/CQ	PDAC Pan 02 cells	GEM 0.5 µg/mL + CQ 2.5 µg/mL for 48 h	↓Viability ↓Migration or invasion ↓Proliferation	↑LC3-II/LC3-I and p62; ↑Autophagosomes ↓Degradation of paxillin and MMP-2	[25]
CQ + IMA	CML K562 cells	IMA 0.25–0.5 µM + 25 µM CQ for 48 h	↑IMA-induced cell death ↓Autophagy	↑LC3-II	[71]
CQ + IMA	IMA-resistant BaF3/E255K and BaF3/T315I lymphoid cells	IMA 5–10 µM + 25 µM CQ for 48 h	↑IMA-induced cell death ↓Autophagy	↑LC3-II	[71]
CQ + IMA	CML K562 cells	IMA 5 µM + CQ 25 µM for 24 h and up to 5 days	↑Sensitivity to IMA ↓Viability ↓Autophagy ↑Necrosis Cell shrinkage	↓Beclin-1 ↑LC3 Nuclei fragmentation	[72]
CQ + IMA	GIST-T1 cells	IMA 1 µM + CQ 50 µM for 72 h or IMA 0.1 µM + CQ 5 µM for 14 d	↓Cell growth ↓Colony formation ↑Apoptosis	↑Caspases 3/7 ↑CC-3 staining	[73]
CQ + IMA	GIST GIST882 cells	IMA 0.5–5 µM for 48 h	↓Cell growth ↑Apoptosis ↓Viability	↓p-ERK/ERK and p-Kit/Kit ↓LC3-II/LC3-I ↑Caspases 3/7	[74]
CQ + Lenvatinib	Papillary thyroid cancer K1 and BCPAP cells	Lenvatinib 10–25 µM + CQ 50 µM for 24 h	↑Inhibitory effect of Lenvatinib ↑Apoptosis ↓Viability and proliferation ↓Angiogenesis	↑LC3-I and LC3-II ↓VEGFA level	[75]
CQ + Apatinib	Anaplastic thyroid cancer KHM-5M and C643 cells	Apatinib 20 µM + CQ 10 µM for 24 h	↓Autophagy ↑Apoptosis	↑LC3-II/LC3-I and p62 ↑Cleaved PARP ↓p-mTOR and p-Akt ↓Autophagosomes	[76]
CQ + Apatinib	Esophageal squamous cell carcinoma ECA-109 and KYSE-150 lines	Apatinib 25 µM + CQ 10 µM for 24 h	↑Apoptosis ↓Autophagy ↓Viability and proliferation ↓Formation of ESCC clones	↑LC3-II/LC3-I and p62 ↑ Bax, ↓Bcl-2, p-Akt, p-mTOR ↓Autophagosomes	[77]
CQ + RAPA	Osteosarcoma MG63 cells	RAPA 20 μM + CQ 20 μM for 24 h	↑Effects of RAPA ↑Apoptosis ↓Proliferation ↓Autophagy	↑LC3-I/II and p62 ↑Cleaved caspases 3 and 9 ↑PARP ↑Autophagosomes	[78]
CQ + RAPA	Human well differentiated liposarcoma 93T449 cells	RAPA 6 µM + CQ 80 µM for 24 h	↓Viability	DNA damage ↑Autophagosomes ↑LC3-II ↑TUNEL-positive cells	[79]
CQ + Salid- roside	Hepatocellular cancer HepG2 and 97H cells	Salidroside 80 µM + CQ 5–20 µM for 48 h	↑Apoptosis ↓Viability ↓Autophagy Changes in cell morphology Chromatin condensation	↑ROS ↓Mitochondrial membrane potential ↑Bax, and cleaved caspase-3 ↓Bcl-2 and Beclin-1 ↑p62, p-mTOR/mTOR, p-PI3K/PI3K, and p-Akt/Akt	[80]
Lys05 + Dacto- lisib	Lung cancer A549 cells	Dactolisib 0.05 µM + Lys05 3.19 µM	↓Autophagy ↑Apoptosis ↓Proliferation	↓*ATG4B*, *LC3A*, *LC3B*, and *KI67* genes ↑*CASP3* ↑LC3B/LC3A and p62	[81]
CQ + Evero- limus	Renal adenocarcinoma A498, RXF393, 769P, and SN12C cells	Everolimus 1.3–19.3 µM + CQ 2.4–19.3 µM for 72 h	Synergic growth inhibition ↑Apoptosis ↓Autophagy	↓Bcl-2 ↓Beclin-1/Bcl-2 complex formation ↓p-4EBP1 and ERK1/2 ↑Caspases 3 and 9	[82]
CQ + Pd(II) complex	Prostate cancer PC-3 and LNCaP cells	Pd (II) complex 12.5 µM + CQ 5 µM for 12–48 h	↓Viability ↑Apoptosis ↓Autophagy ↑ROS	↑Caspases 3/7 ↓Atg5, Beclin-1, LC3, and p62 ↓p-Akt/p-mTOR, p-STAT5, and p-CREB	[83]
CQ + Tamoxifen	Antiestrogen-resistant breast carcinoma MCF7-RR, LCC9 cells	1 μM CQ, 10–1000 nM Tamoxifen for 6 days	↓Cell growth ↓ Autophagy ↑Cell death	↑Autophagosomes ↑LC3-II and p62	[84]
CQ + Faslodex	Antiestrogen-resistant breast carcinoma MCF7-RR, LCC9 cells	1 μM CQ, 10–1000 nM Faslodex for 6 days	↓Cell growth ↓ Autophagy ↑Cell death	↑Autophagosomes ↑LC3-II and p62	[84]
CQ + Ipata-sertib	MDAMB231, MDAM468, MCF7, and SKBR3 breast cancer cell lines	Ipatasertib 1–10 μM + CQ 1–10 μM	↑Apoptosis ↓Autophagy ↓Proliferation ↓Clonogenic capacity ↓Spheroid-forming capacity	↑Cleaved PARP ↑LC3-II and p62 ↑Autophagosomes	[85]
CQ + Taselisib	MDAMB231, MDAM468, MCF7, and SKBR3 breast cancer cell lines	Taselisib 1–10 μM + CQ 1–10 μM	↑Apoptosis ↓Autophagy ↓Proliferation ↓Clonogenic capacity ↓Spheroid-forming capacity	↑Cleaved PARP ↑LC3-II and p62 ↑Autophagosomes	[85]
CQ + IR	Glioblastoma no. 993, no. 1095 and G112SP cells	CQ 30 μM + IR 2.5 Gy for 72 h	↓Proliferation ↑Cell death Cell cycle arrest	↑LC3B-II and p62 ↓Akt and Ki67 ↑SubG1 population	[30]
CQ + Vemu- rafenib	Glioblastoma 794 and AM38 cells	Vemurafenib 1 μM + CQ 5 μM	↓Clonogenic growth		[37]
CQ + Trame tinib	Glioblastoma 794 and AM38 cells	Trametinib 7.5–30 nM + CQ 5 μM	↓Growth ↓Clonogenic growth		[37]
CQ + Vemu- rafenib	Patient-derived glioblastoma cells	Vemurafenib 1–2 μM + CQ 10–20 μM for 72 h	↓Autophagy ↓Tumor growth	↑LC3B-II, p-ERK/ERK ↑Caspases 3/7 ↓p-Akt and pS6	[37]
CQ + Sorafenib	Thyroid cancer TPC1, ACT1, and KTC1 cell lines	Sorafenib 100 nM + 50 μM CQ for 48 h	↑Apoptosis ↓Autophagy	↑LC3B-II and p62	[41]
HCQ + Temozo-lomide	Glioblastoma U-87 Mg cells	TMZ 100 µg/mL + HCQ 1 µg/mL for 24 h	↑Apoptosis ↓Autophagy	↑LC3-II ↑ROS	[65]
CQ + PTX + Apatinib	Esophageal carcinoma ECA-109 and KYSE-150 cells	PTX 5 μM + CQ 10 μM + Apatinib 25 μM for 24–72 h	↑Sensitivity to PTX ↑Apoptosis ↓Proliferation ↓Colony formation	↑Bax and cleaved caspase-3 ↓Bcl-2, p-Akt, and p-mTOR	[77]

Abbreviations: DOX—doxorubicin, PTX—paclitaxel, CIS—cisplatin, CPT—carboplatin, OXP—oxaliplatin, GEM—gemcitabine, IMA—imatinib, RAPA—rapamycin, IR—irradiation, CSCs—cancer stem cells, TNBC—triple-negative breast cancer, GSCs—glioblastoma stem-like cells, HUVECs—human umbilical vein endothelial cells, PDAC—pancreatic duct adenocarcinoma cells, CML—chronic myeloid leukemia, GIST—gastrointestinal stromal tumor cells. Comments: ↑—increased expression, ↓—downregulation

### 4.2. Chloroquine and Paclitaxel (PTX)

Paclitaxel, a tricyclic diterpenoid belonging to taxanes and found in the bark and needles of *Taxus brevifolia*, is one of the most successful natural chemotherapeutic compounds [86,87]. Due to minimal toxicity, high efficiency, and broad-spectrum antitumor activity, PTX is widely used for the therapy of ovarian, cervical, breast, colorectal, esophageal, lung, and prostate cancer, either alone or in combination with other agents. The major mechanism of its activity is a capacity to disrupt microtubule-assembling dynamics and induce cell cycle arrest at the G2/M phase, leading to apoptosis. However, as for other chemotherapeutic drugs, a major problem of PTX application is the development of chemoresistance due to protective autophagy [88].

The synergic effects of CQ and PTX in suppressing viability and growth were accompanied by the inhibition of autophagy in MCF-7 human breast tumor cells [39] and three TNBC cell lines [32]. Moreover, CQ increased the sensitivity to PTX and reduced lung metastases, tumor growth, and recurrence in orthotopic murine MDAMB231 and SUM159PT tumor models and diminished the CD44^+^/CD24^−/low^ CSC population in a clinical trial [32]. The co-exposure of esophageal carcinoma EC109 cells to CQ and PTX was found to enhance the suppressive effect of PTX by inhibiting autophagy through the Akt/mTOR pathway [89]. A phase II clinical trial, which recruited patients with advanced or metastatic breast cancer (of HR^+^/HER2^−^ and TNBC types) who previously did not benefit from anthracycline-based chemotherapy, has shown that CQ in combination with taxane or taxane-like agents (paclitaxel, docetaxel, nanoparticle (NP) albumin-bound nab-paclitaxel, and ixabepilone) increases the objective response rate in comparison to that expected for PTX-based therapy itself, with good tolerance and a low rate of adverse effects [90] (Table 3).

### 4.3. Chloroquine- and Platinum-Based Anticancer Drugs

The cohort of clinically approved platinating derivatives includes cisplatin (CIS), carboplatin (CPT), and oxaliplatin (OXP). The major mechanism of their action is DNA damage followed by the inhibition of transcription, but they are also able to exert cytoplasmic effects such as mitochondrial damage, ER stress, the suppression of ribosome biogenesis, and the elevation of micro-RNA activity [91,92]. They are widely used as a first-line chemotherapy compound for ovarian, cervical, testicular, bladder, esophageal, lung, and head and neck cancers; brain tumors; and neuroblastoma. However, the resistance and many side effects (nephrotoxicity, neurotoxicity, and hepatotoxicity) of these agents are reported, which drives the necessity to reduce their toxicity [93].

*Cisplatin.* CQ enhanced the sensitivity to CIS treatment in endometrial adenocarcinoma cells [35], thyroid cancer cell lines (TPC1, ACT1, and KTC1) [41], and SH-SY5Y cells [63]. In all of these cells, CQ effects were associated with the suppression of autophagy accompanied by increased LC3 and p62 expression. In epithelial ovarian cancer SKOV3 and hey cells, CQ alone had no effect on tumor migration and invasion capacities but alleviated CIS-induced autophagy with an upregulation of apoptosis-related proteins [64]. In mice bearing a gastric cancer xenograft, CQ enhanced CIS chemosensitivity and the antitumor effect via the downregulation of multidrug resistance gene MDR1/P-gp and activation of caspase-3, as well as via the inhibition of CIS-triggered autophagy [94]. In a mouse hepatocarcinoma xenograft model, CIS or CQ alone was able to reduce tumor growth; however, their combination significantly augmented the antitumor effect and impaired the proliferation of tumor cells by causing a higher level of apoptosis [95]. The inhibition of autophagy with HCQ and CIS enhanced apoptosis and potentially therapeutic oxidative stress in neuroblastoma SH-SY5Y [65].

*Carboplatin.* In combination with CPT, CQ exerted an additive antitumor effect in TNBC SUM159 stem cells and effectively reduced the growth of mice CPT-resistant SUM159 orthotopic xenografts proven to be linked with the inhibition of CPT-induced autophagy [33]. The effectiveness of the CQ/CPT combination was confirmed in experiments on epithelial ovarian tumor cells from patients and mice xenografts, in which such a treatment decreased the CSCs pool, with surface co-expression of CD117 (c-Kit) and CD44, and suppressed their tumorigenic potential and spheroid-forming ability [38]. In heavily pretreated patients with advanced solid tumors of different origin (GIST, neck and head, colorectal, urothelial, esophageal, etc.), a combination of CQ or HCQ with CPT increased progressive-free disease and overall survival (OS), although some side effects were reported [96]. Importantly, in the exosomes obtained from the blood plasma of patients who received such treatment, both LC3-B isoforms were detected at advanced time points of the second and third cycles [97].

*Oxaliplatin.* Apoptotic cell death induced by OXP was significantly enhanced by CQ treatment in hepatocellular carcinoma HepG2 cells with ATG7 knockdown due to the inhibition of autophagy [66]. The application of CQ sensitized a few colon cancer cell lines to OXP under both oxic and hypoxic conditions and showed a synergistic interaction in suppressing the growth of mice HT29 xenografts with a reduced number of autophagosomal cells [67]. Recently, biomimetic nanoparticles encapsulating both HCQ and OXP were shown to reduce the tumor capacities of hepatocellular carcinoma cells in vitro and in vivo by blocking or reversing autophagy [68].

### 4.4. Chloroquine and Gemcitabine (GEM)

Gemcitabine is a nucleoside metabolic inhibitor whose active metabolites function as deoxycytidine analogs able to replace the building blocks of nucleic acids during DNA elongation, thus preventing DNA synthesis, arresting tumor growth, and promoting apoptosis [98]. Although GEM was initially approved for the treatment of pancreatic cancer, it is currently used as an adjunct therapy for various solid tumors, such as ovarian cancer, non-small-cell lung carcinoma, and metastatic breast cancer. However, the resistance to GEM remains a serious problem among a noticeable rate of patients. It is not surprising that CQ was tested as a potential synergist to GEM.

In vivo CQ and GEM co-exposure more effectively eliminated tumors and improved the overall survival of mice bearing pancreatic patient-derived PDAC xenografts via the inhibition of the CXCL12/CXCR4 pathway with reduced phosphorylation of downstream effectors ERK and STAT3 and inhibition of hedgehog signaling [27]. The addition of CQ strengthened the cytotoxic effects of GEM in human gallbladder cancer cells (GBCs) in vitro and inhibited the growth of GBC xenografts in mice in vivo, with an upregulation of the LC3-II/LC3-I ratio and Bax, downregulation of Bcl-2 and PARP, and inhibition of the Akt/mTOR pathway [69]. The GEM/CQ combination significantly reduced the viability of human pancreatic cancer PANC-1 cells, although CQ alone did not exert any effect [70]. The addition of CQ or HCQ to GEM therapy increased the OS of patients with advanced solid tumors of different types who previously received other treatment regimens [96].

As for other chemotherapy drugs, new delivery strategies with enhanced penetration ability have been developed. The combined delivery of GEM and poly lactic-co-glycolic acid (PLGA) nanoparticles loaded with CQ, created as carriers to reduce its doses, to mice bearing orthotopic pancreatic cancer xenografts diminished tumor progression and suppressed the density of activated tumor cells at lower CQ doses [99]. Chen et al. [100] designed pH-sensitive PDGL-GEM@CAP/CQ particles consisting of GEM loaded in 6PA-modified DGL and co-precipitated with CQ and calcium phosphate. The administration of these particles to cultured pancreatic Pan 02 cells or mice bearing Pan 02 xenografts intensified antitumor GEM/CQ effects via the inhibition of proliferation, tumor growth, metastases and fibrosis, suppression of autophagy, and a decrease in the number of activated fibroblasts. In contrast to GEM monotherapy, adjuvant autophagy inhibition with HCQ significantly increased the median OS and DFS of the patients with high-risk PDAC [101].

**Table 3:** The effects of single CQ treatment or combination with chemotherapy drugs on animal tumor xenografts models.

Agent	Experimental System	Treatment Regime	Effect	Molecular Markers	Reference
CQ	Glioblastoma U87MG xenografts of NMRI nude mice	CQ 30 mM/day intracranially for 17 days	↓Tumor growth ↓Cell viability ↓Number of mitotic cells		[25]
CQ	Melanoma SKMel23 cells xenografts of NOD-SCID mice	CQ 25 mg/kg (IP) twice/week for 3 weeks	↓Tumor growth ↓Autophagy		[26]
CQ	Immunocompromised mice implanted with patient-resected PDAC cells	CQ 50 mg/kg (IP) for 21 days	↓CSCs-driven metastases ↓Tumorigenicity	↓CD133+ cells number ↓*ALK4* ↓Nodal/Activin ↓Self-renewal genes	[27]
CQ	Liver cancer HepG2-GFP xenograft of nude mice	CQ 80 mg/kg twice daily 3 d on/2 d off (SC) for 25 days	↓Tumor growth and weight ↓Proliferation	↓Ki-67 ↑cleaved PARP	[28]
CQ	Athymic nude mice with orthotopic MDAMB231 breast cancer tumor	CQ 10 mg/kg daily (IP) for 2 2 weeks	↓Tumor growth ↓Lung metastasis	↓CD44^+^/CD24^−/low^ stem cells number	[32]
CQ HCQ	Immunodeficient NOD/Shi-scid/IL-2Rγnull (NOG) mice transplanted with ATLL MT2 or Su9T01 cells	CQ 50 mg/kg/day (IP) or HCQ 6.5–60 mg/kg/day (OR) for 21 days	↑Survival ↓Tumor growth and weight Degeneration and necrosis of tumor cells	↑Caspase-3 ↑Condensed hyperchromatic or fragmented nuclei with shrunken cytoplasm	[34]
CQ	Female BALB/c mice with MCF-7 xenograft	CQ 50 mg/kg (IP) once/3 days for 43 days	↓Viability and growth ↑Apoptosis ↓Autophagy	DNA damage Cytochrome C release ↑Bax and p53 ↑Caspases 3 and 9	[40]
CQ + DOX	Female BALB/c mice with MCF-7 xenograft	DOX 2 mg/kg (IP) + CQ 50 mg/kg (IP) once/3 days for 43 days	↓Tumor growth, ↑Apoptosis ↓Autophagy	DNA damage ↑Autophagosomes Cytochrome C release ↑Bax, p53, caspases 3 and 9, Beclin-1, ATG7, LC3-II, and p62 ↓PI3K, Akt, mTOR, and Bcl-2	[40]
CQ + DOX	Female mice injected with Ehrlich ascites carcinoma (EAC) cells	DOX 1.5 mg/kg and 3 mg/kg + CQ 25 mg/kg and 50 mg/kg (IP) at 2, 7, and 12 days	↓Disruption of alveolar structure ↓Oxidative stress	↓MDA, CAT, GPx, SOD, iNOS, and eNOS ↑ NGAL	[53]
CQ + PEG-DOX+ pUH	BALB/c mice subcutaneously injected with 4T1 breast tumor cells	PEG-DOX 10 mg/kg (IV) + CQ 50 mg/kg + 15 min on-tumor pUH on day 5 after tumor implantation up to 60 days	↓Viability ↓Tumor growth ↑ Animal survival	DNA damage ↑LC3-II ↑TUNEL-positive cells	[58,59]
CQ + DOX. HCl in DA-DIV/C nanovesicles	Female BALB/c nude mice subcutaneously inoculated with DOX-resistant K562/ADR cells	DOX-HCl 5 mg/kg + CQ 10 mg/kg (IV) at 0, 2, 4, and 6 days	↓Tumor volume and weight ↓Autophagy ↓Cell density ↑Necrosis DNA damage	↓Ki67 ↑TUNEL-positive cells ↑LC3-II	[62]
CQ + PTX	Athymic nude mice with orthotopic MDAMB231 and SUM159PT tumors	PTX 15–30 mg/kg (IP) weekly + CQ 10 mg/kg daily for 2 weeks or twice/week for 4 weeks	↑Sensitivity to PTX ↓Tumor growth ↓Lung metastasis ↓Tumor recurrence ↓PTX-induced CSCs population	↓CD44^+^/CD24^−/low^ CSCs	[32]
CQ + CIS	Nude mice with ovarian cancer SKOV3 xenograft	CIS 5 mg/kg/6 days + CQ 60 mg/kg/day (IP) for 21 days	↓Tumor volume and weight	↑Cleaved caspase-3 ↓Ki-67-positive cells	[64]
CQ + CIS	Nude BALB/C female mice with gastric cancer SGC7901 xenograft	CIS 5 mg/kg + CQ 45 mg/kg every three days 10 times	↓Tumor weight	↓LC3II/I ratio and Beclin-1 ↓MDR1/P-gp ↑caspase-3	[94]
CQ + CIS	BALB/C nude mice with hepatocarcinoma SMMC-7721 xenograft	CQ 60 mg/kg + CIS 3 mg/kg (IP) thrice/week for 2 weeks	↓Tumor volume and weight ↑Apoptosis ↓Proliferation	DNA damage ↓Ki-67-positive cells	[95]
CQ + CPT	Immunodeficient SCID-Beige mice with TNBC SUM159 xenograft	CPT 24 mg/kg weekly + CQ 30 mg/kg every 3 days for 3 weeks	↓Tumor volume ↓Viability ↑Apoptosis	↓Mitochondrial metabolic activity ↓Bcl-2, Rad50, Rad51 ↑LC3B-II, and p62	[33]
CQ + CPT	Immunodeficient NSG mice injected with CD45^-^CD44^+^ epithelial ovarian tumor cells	CPT 50 mg/kg + CQ 100 mg/kg every 2 days weekly for 16 weeks	↓Tumor volume	↓CD44^+^/CD117^+^ cells population ↓Ki67	[38]
CQ + OXP	Immunodeficient C/.B.17 SCID mice injected with colon cancer HT29 cells	OXP 5 mg/kg (IP) per week for 2 weeks + CQ 3.5 mg/kg daily for 21 days	↓Tumor growth and volume ↓Autophagosomal cells	↓LC3 staining	[67]
TH-NP with HCQ + OXP	Nude mice with hepatocellular carcinoma HCCLM3 xenograft	OXP 10 mg/kg + HCQ 20 mg/kg (IV) every three days for 30–49 days	↓Tumor growth ↓Metastases ↓Autophagy	↑Cleaved caspase 3 and PARP ↓Ki67 ↓Autophagosomes/ autolysosomess	[68]
CQ + GEM	Immunocompromised mice implanted with patient-resected PDAC	GEM 125 mg/kg (IP) for 52 days + CQ 50 mg/kg (IP) for 21 days	↓Tumor growth ↑Survival rate	↓ CD133+ CSCs ↓Nodal/Activin pathway	[27]
CQ + GEM	Male BALB/c nude mice injected with gallbladder cancer SGC-996 cells	GEM 20 mg/kg (IP) + CQ 60 mg/kg (IP) twice/week for 22 days	↑Sensitivity to GEM ↓Tumor growth		[69]
CQ-loaded PLGA nanoparticles + GEM	BALB/c AJcl nu/nu female mice orthotopically transplanted with immortalized patient- derived pancreatic stem cells and SUIT-2 cancer cells	GEM 40 mg/kg (IV) on days 10, 17, and 24 + Nano-CQ 30 mg/kg (IV) on days 10, 17, and 24	↓Density of activated cancer stem cells ↑Sensitivity to GEM ↓Tumor volume and weight	↓αSMA	[99]
PDGL-GEM@CAP/ CQ	Mice bearing pancreatic cancer Pan 02 xenografts and orthotopic pancreas Pan 02 tumor	GEM 3 mg/kg (IV) + CQ 15 mg/kg (IV) every other day 4 times	↓Tumor growth ↓Metastases ↑Tumor necrosis ↓Number of activated fibroblasts ↓Fibrosis ↓Autophagy	↑Autophagosomes ↑LC3II/LC3I ratio and p62 ↓MMP-2, IL-6 ↓Collagen ↑Paxillin ↓αSMA	[100]
CQ + IMA	Female athymic nude NMRI nu/nu with heterotopic GIST-T1 xenograft	IMA 50 mg/kg (OR) twice/day + CQ 60 mg/kg (IP) daily for 15 days	↑Apoptosis No effect on tumor growth	↑CC-3 staining	[73]
CQ + IMA	NOD/SCID male mice implanted with IMA- sensitive and resistant GIST882 cells	IMA 150 mg/kg (OR) twice/day + CQ 60 mg/kg (IP) daily for 28 days	↓Autophagy No effect on tumor growth	↑LC3II ↓p-ERK/ERK	[74]
CQ + Lenvatinib	Nude mice injected with thyroid cancer K1 cells	Lenvatinib 30 mg/kg + CQ 50 mg/kg for 14 days	↑Anticancer LEN effect ↓Tumor growth ↓Angiogenesis	↓VEGFA, CD31, and C-Myc	[75]
CQ + Lenvatinib	Nude BALB/c mice injected with hepatocellular carcinoma HCCLM3 cells	Lenvatinib 5–10 mg/kg (IP) + HCQ 50 mg/kg (IP)	↓Tumor growth ↓Lung metastases ↑Overall survival		[102]
CQ + Apatinib	Male BALB/c nude mice injected with KHM-5M thyroid cancer cells	Apatinib 50 mg/kg (OR) daily + CQ 60 mg/kg (OR) daily for 26 days	↓Tumor volume and weight ↓Proliferation ↑Apoptosis	↑Cleaved caspase-3 ↑TUNEL-positive cells ↓Ki67	[76]
CQ + Apatinib	Male BALB/c nude mice injected with esophageal carcinoma ECA-109 cells	Apatinib 60 mg/kg OR) daily + CQ 60 mg/kg (OR) daily for 4 weeks	↓Tumor volume and weight ↓Proliferation ↑Apoptosis	↑Cleaved caspase-3 ↑TUNEL-positive cells ↓Ki67-positive cells	[77]
CQ + RAPA	Athymic nude mice injected with patient- derived dedifferentiated liposarcoma	RAPA 1 mg/kg/day (IP) + CQ 100 mg/kg/day (IP) for 15 days	↓Tumor growth ↓Cancer cells density ↑Apoptosis	↑TUNEL-positive cells	[103]
CQ + Salid- roside	Female BALB/c mice subcutaneously injected with HepG2 cells	Salidroside 80 mg/kg (IP) + CQ 5 mg/kg (IP) every other day for 4 weeks	↓Tumor growth ↓Number of tumor cells	↑Bax ↓Bcl-2	[80]
CQ + 5-FU	BALB/c nude mice with hepatocarcinoma SMMC-7721 xenograft	5FU 30 mg/kg (IP) + 60 mg/kg CQ (IP) trice/week for 2 weeks	↑Sensitivity to 5-FU ↑Apoptosis ↓Proliferation ↓Tumor growth	↑TUNEL-positive cells ↓Ki67-positive cells	[95]
CQ + Tamo- xifen	Athymic nude mice injected with breast cancer MCF7-RR or LCC9 cells	Tamoxifen 32 mg/kg/d + CQ 1–2 mg/mouse/d (OR) for 5 weeks	↓Tumor growth ↑Angiogenesis ↓Macrophage activation	↑CD31-positive cells ↑pVEGFR2 ↑CD68-positive cells	[84]
CQ + Fas- lodex	Athymic nude mice with breast cancer MCF7-RR or LCC9 xenografts	Faslodex 0.5 mg/mouse/w (SC) + CQ 1–2 mg/mouse/d (OR) for 5 weeks	↓Tumor growth ↑Angiogenesis	↑CD31-positive cells ↑pVEGFR2	[84]
CQ + Tase-lisib	Female NOD/SCID athymic mice injected With TNBC MDAMB231 cells	Taselisib 5 mg/kg (OR) 5 days/week + CQ 30 mg/kg (OR) 5 days/week for 2 weeks	↑Antitumor PTX effect ↓Tumor growth		[85]
CQ + Nelfi- navir + RAPA + Dasatinib + Metformin	Female Nu/nu mice subcutaneously injected with cisplatin-resistant ovarian cancer OVCAR3 cells	CQ 30 mg/kg + Nelfinavir 250 mg/kg + RAPA 2.24 mg/kg + Dasatinib 4 mg/kg + Metformin 150 mg/kg in 50% PEG400 for 7 days	Tumor remission	↑ LC3B-II and Grp78	[104]
CQ + Apatinib + PTX	Nude BALB/c mice injected with esophageal carcinoma ECA-109 cells	Apatinib 60 mg/kg (OR) daily + CQ 60 mg/kg (OR) daily + PTX 15 mg/kg (IP) twice/week for 4 weeks	↓Tumor volume and weight ↑apoptosis ↓Proliferation ↑Apoptosis	↑Cleaved caspase-3 ↑TUNEL-positive cells ↓Ki67	[77]
CQ + Tase- lisib + PTX	Female NOD/SCID athymic mice injected With TNBC MDAMB231 cells	Taselisib 5 mg/kg (OR) 5 days/week + CQ 30 mg/kg (OR) 5 days/week + PTX 10 mg/kg IP once/week for 2 weeks	↑Antitumor effect of PXT and Taselisib ↓Tumor volume and weight		[85]
CQ + IR	Female NMRI immunodeficient mice injected with GBCs no. 993, no. 1095 and G112SP cells	CQ 14 mg/kg IP IR 2.5 Gy for 6 days	↑Survival ↑Sensitization to IR		[30]

Abbreviations: IP—intraperitoneally, SC—subcutaneously, OR—orally, IV—intravenously. ↑—increased expression, ↓—downregulation.

### 4.5. Chloroquine and Tyrosine Kinase Inhibitors

*Imatinib (IMA).* Imatinib is a small molecule tyrosine kinase inhibitor targeting numerous enzymes like CSF1R, c-KIT, FLT3, and platelet-derived growth factor receptor PDGFR-β, but it is reasonably selective to BCR-ABL fusion protein. It binds to the ATP pocket at a kinase active site, thus preventing the downstream phosphorylation of target proteins. IMA is the most common first-line cytotoxic agent for the treatment of chronic myeloid leukemia (CML) and gastrointestinal stromal tumor (GIST) in systemic therapy, but CML stem cells are intrinsically resistant to IMA [105,106].

An important role of autophagy in the resistance of CML cells to IMA was established in K562 cells, in which CQ or IMA alone did not change the rate of death while CQ/IMA co-treatment enhanced the sensitivity to IMA and accelerated apoptotic cell death. Moreover, the combination of these drugs produced the same effects in IMA-resistant lymphoid cell lines [71]. CQ potentiated IMA-induced cytotoxicity and reduced the long-term viability of K562 cells due to the inhibition of autophagy initiation and autophagosome turnover [72]. In GIST-T1 cells treated with CQ as a single agent or in combination with IMA, the suppressed growth and decreased viability were accompanied by increased LC3-II levels. Furthermore, treatment with IMA/CQ increased apoptosis in a mouse GIST-T1 xenograft [73]. Although CQ or IMA alone did not inhibit or weakly inhibited the growth of GIST882 IMA-resistant cells, CQ addition enhanced the suppressive effect of IMA on cell proliferation and promoted apoptosis by blocking autophagy and altering the level of ERK phosphorylation [74]. A phase II clinical trial, however, did not reveal any pronounced differences in long-lasting (12 and 24 months) “success” rates after 48-week administration of IMA/CQ, although the authors noticed some molecular responses [107].

*Lenvatinib*. Lenvatinib is a potent tyrosine kinase inhibitor targeting PDGFRα, vascular endothelial growth factor receptors VEGFR1-3, fibroblast growth factor receptors FGFR1-4, tyrosine kinase receptor c-Kit, and RET proto-oncogene. It is widely used for the treatment of thyroid cancer and hepatocellular carcinoma [108,109]. Although the resistance and side effects following its application are common, data on Lenvatinib and CQ therapy are scarce. The effectiveness of CQ/Lenvatinib co-exposure was shown in thyroid cancer K1 and BCPAP cells, with the suppression of Lenvatinib-induced autophagy leading to the inhibition of proliferation and angiogenesis, increased apoptosis, and reduced VEGFA levels, while the co-treatment of mice bearing a K1 xenograft diminished tumor growth accompanied by decrease in VEGF markers VEGFA and CD31 and proliferation marker c-Myc [75]. Combined HCQ/Lenvatinib therapy increased the overall survival of mice with hepatocellular carcinoma xenografts accompanied by the inhibition of tumor growth and lung metastases [102].

*Apatinib.* Apatinib is a tyrosine kinase inhibitor that selectively inhibits VEGFR2 and has a mild activity towards c-Kit and c-SRC tyrosine kinases [110]. The major anticancer effect of Apatinib is the blockage of angiogenesis, namely VEGF-mediated endothelial cell migration and proliferation leading to the suppression of new blood vessel formation in tumor tissue. The inhibition of Apatinib-induced autophagy with CQ in vitro increased apoptosis in thyroid cancer KHM-5M and C643 cells through the downregulation of p-Akt and p-mTOR, while Apatinib/CQ therapy augmented the suppression of the mice thyroid cancer xenograft in vivo [76]. In ECA-109 and KYSE-150 esophageal squamous carcinoma cells, CQ administration enhanced the anticancer effects of Apatinib in vivo and in vitro by inhibiting autophagy via the IRE-1α–Akt–mTOR pathway and enhancing apoptosis via the stimulation of Bax and caspase-3 but decreasing the levels of Bcl-2 [77].

### 4.6. Chloroquine and PI3K/Akt/mTOR Inhibitors

The PI3K/Akt/mTOR (phosphoinositide 3-kinase/Akt kinase/mammalian target of rapamycin) cascade is one of the most crucial signaling pathways controlling key cellular functions such as proliferation, growth, metabolism, and survival. Since its abnormal activation is a frequent event in many human malignancies, while the suppression leads to an upregulation of autophagy, the combination of PI3K/Akt/mTOR and autophagy inhibitors was suggested to have a higher therapeutic benefit [111,112,113]. To date, more than 40 different agents targeting this pathway have been tested in various stages of clinical trials, but only a few of them have been approved for cancer therapy.

In MG63 osteosarcoma cells, CQ enhances apoptotic cell death promoted by mTOR inhibitor rapamycin (RAPA) by blocking the activity of downstream molecules of Akt/mTOR pathway 4E-BP1 and p70S6k, increasing the expression of autophagy-related proteins LC3-II and Atg12-Atg5, and decreasing the p62 level [78]. Although CQ was not effective as a single treatment, CQ/RAPA exposure induced apoptosis via the overaccumulation of autophagosomes in well-differentiated human liposarcoma (WDLS) 93T449 cells [79] and arrested the growth of dedifferentiated liposarcoma in mice bearing patient-derived orthotopic xenografts (DDLS PDOX) [103].

The addition of CQ to Salidroside, a glycoside isolated from the root of *Rhodiola rosea* L., enhanced the sensitivity of hepatocellular cancer HepG2 and 97H cells to this compound and exerted a synergic effect on the growth of the mice HepG2 xenograft by suppressing the invasion and metastasis of cancer cells through the PI3K/Akt/mTOR pathway, promoting mitochondrial dysfunction and altering the ratio between the expression of pro- and anti-apoptotic proteins [80,114]. The combination of imidazoquinoline derivative Dactolisib, dual PI3K/*mTOR* inhibitor*,* and Lys05, dimeric CQ compound, exerted a significant additive effect in the cultured lung cancer A549 cells via the stimulation of apoptotic genes, downregulation of proliferative gene marker *KI67,* and blocking the expression of autophagic genes [81]. In a few renal cancer cell lines, the synergic effects of CQ and Everolimus, RAPA analog approved for second-line therapy, included the suppression of cell viability, inhibition of autophagy, and shift to apoptosis via the intrinsic mitochondrial pathway associated with a decrease in the Beclin-1/Bcl-2 complex, although the tested cell lines had different sensitivities to such treatment [82]. A phase I/II clinical trial that recruited patients with previously treated clear-cell renal carcinoma (ccRCC) showed that combined HCQ/Everolimus therapy is safe and tolerable and led to a partial response and prolonged stable disease in a subset of patients, although the mutations in the mTOR signaling pathway were associated with shorter survival [115]. A significant antitumor capacity of HCQ combined with Temsirolimus, an intravenous RAPA analog, due to the modulation of autophagy was reported in a phase I clinical trial in patients with solid tumors and melanoma [116].

### 4.7. Chloroquine and Other Agents

In PC-3 and LNCaP prostate cancer cell lines, combined treatment with the Palladium (Pd)(II) complex and CQ caused pyknotic nuclei and induced apoptosis accompanied by increased activity of caspase 3/7. Moreover, in PC-3 cells, such exposure downregulated autophagy proteins Atg5, Beclin-1, and LC3, pro-survival PI3K/Akt/mTOR-related protein, and Jak/STAT5, while p38 was highly phosphorylated [83]. The study of Cook [84] has shown that CQ addition augmented the sensitivity of breast cancer cells resistant to endocrine therapies to estrogen receptor-α (ERα)-targeted agents Tamoxifen or Faslodex both in vitro (in MCF7-RR, LCC9, and ZR-75-1/ICI-R cells) and in vivo (in mice xenograft models), with this effect linked with alterations in immune response. CQ supplementation inhibited autophagy and enhanced the cytotoxic effect of Sorafenib in TPC1, ACT1, and KTC1 thyroid cancer cell lines [41]. The suppression of autophagy with CQ was able to improve the responses of the cultured brain tumor cells resistant to BRAF blockers to chemotherapy with MEK inhibitor Trametinib and, more importantly, reduce the metastases of brain glioblastoma in patients with BRAF mutations [37]. HCQ enhanced apoptosis and potentially therapeutic oxidative stress in glioblastoma U-87 cells treated with Temozolomide, which possesses an ability to alkylate/methylate DNA, thus triggering its damage and the death of tumor cells [65]. The combination of 5-FU with CQ significantly reduced the viability of a human pancreatic cancer PANC-1 cell line in comparison to a single 5-FU exposure, although CQ alone did not exert any effect [70]. In a mouse xenograft hepatocarcinoma model, 5-FU or CQ alone was able to reduce tumor growth. However, their combination significantly augmented the antitumor effect and impaired the proliferation of tumor cells by causing a higher level of apoptosis [95]. A few randomized clinical trials that attempted to use CQ as an adjuvant for conventional chemotherapy and radiotherapy of patients with glioblastomas (GBM) reported an enhanced response to antineoplastic treatment and improved mid-term survival [117,118]. A recent meta-analysis of clinical trials allowed the authors to conclude that CQ supplementation led to significantly improved survival or remission time and decreased mortality, with a low incidence of adverse effects and seizures, thus showing some effectiveness in the treatment of glioblastoma [119]. A broad range of responses, from minor to partially good, and stable disease were reported in a study evaluating the effects of therapy with a combination of HCQ and Bortezomib, a reversible inhibitor of the chymotrypsin-like subunit of the 26S proteasome, in a group of patients with relapsed or refractory myeloma [120].

### 4.8. Chloroquine in Multi-Drug Combinations

The development of chemoresistance and the existence of mutations have forced the search for new treatment combinations consisting of drugs acting on different cellular targets. In many of such combinations, CQ was added to suppress cytoprotective autophagy. In TNBC MDAMB231 or MDAMB468 cells, CQ potentiated the antitumor effect of the combined addition of PTX and PI3K/Akt/mTOR inhibitors Ipatasertib and Taselisib by reducing autophagic flux and enhancing apoptosis [85]. In breast cancer MDAMB231 and MCF-7 cells, a triple combination of CQ, DOX, and Ixazomib, which binds the β5 subunit of the 20S proteasome, thus inhibiting its chymotrypsin-like activity*,* synergistically suppressed cell growth and increased the sensitivity to chemotherapy [121]. Using COAST (combination of autophagy selective therapeutics: CQ, Nelfinavir, RAPA, Dasatinib, and Metformin in 50% PEG400), Delaney et al. [104] showed that this drug cocktail effectively arrested the growth of three types of mice xenografic ovarian cancers resistant to CIS-Docetaxel chemotherapy, with residual tumors exhibiting enhanced levels of LC3-II and ER stress marker GRP78. The combined addition of Apatinib and CQ enhanced the anti-proliferative effect of PTX on esophageal squamous carcinoma cells ECA-109 and KYSE-150 in vitro or intensified tumor suppression in vivo [77]. A modest improvement in clinical responses (higher ORR and PFS) following combined HCQ/CPT/PTX therapy was reported in a study that recruited patients with newly diagnosed stage IV non-small-cell Kras-mutated lung cancer [122]. Preoperative HCQ plus GEM/nab-PTX chemotherapy of the patients with potentially resectable pancreatic adenocarcinoma demonstrated an improved Evans grade histopathological response, decreased CA19-9 tumor marker level correlated with enhanced OS, and increased immune cell infiltration within the tumor [123]. However, the addition of HCQ to conventional chemotherapy improved the histopathological response rate, but not OS, of patients suffering from PDAC with loss of tumor suppressor SMAD4 [124] or patients with metastatic PDAC [125].

## 5. Conclusions

**Figure 3:**
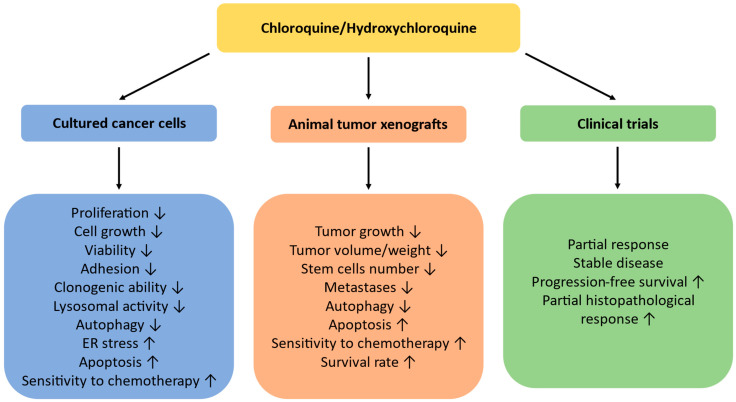
Anticancer effects of CQ/HCQ in experimental studies and in clinical trials. ↓—inhibition, ↑—enhancement.

Overall, the majority of experimental in vitro and in vivo works has shown that the addition of CQ or HCQ to conventional cytotoxic drugs significantly enhanced their anticancer effects, especially in cultured cells (Figure 3). Therefore, these agents can be suggested as effective adjuvant agents sensitizing cancer cells to chemotherapy and offering more efficient elimination of tumors, which can improve clinically relevant curative rates. However, the clinical trials were not always successful, with a “partial response” being the most frequent finding. Some trials did not reveal any significant increase in overall survival rates, probably due to the enrollment of patients with advanced stages of diseases or the existence of undetected mutations. Another weakness of many clinical trials is the absence of control groups of patients, where the conclusions have been made based on the “expected survival rate”. Moreover, long CQ and HCQ exposure is known to be associated with serious adverse effects such as allergic reactions, irreversible retinal toxicity, gastrointestinal discomfort, cardiomyopathy symptoms, neuromyotoxicity, and bone marrow suppression [126]. Moderate side effects linked with their application have been observed in almost all clinical trials listed in Table 4. Finally, the effects of CQ and HCQ appear to be cancer-specific, and they do not exclusively inhibit autophagy, which raises some pessimism regarding their use. Nevertheless, they should be further tested in experimental and clinical settings with malignancies of different origins to reveal the types of tumors most sensitive to such treatment and the most effective chemotherapeutic combinations. To more precisely target autophagy and diminish possible side effects, the development of new more specific and potent autophagy inhibitors is required. 

**Table 4:** CQ or HCQ and chemotherapy drugs in clinical trials.

Agents	Tumor Type	Concentration	Effects	Reference
CQ + PTX, nab-PTX, Docetaxel, or Ixabepilone	Advanced or metastatic anthracycline- refractory breast cancer	CQ 250 mg (OR) daily + PTX 80–175 mg/m^2^ (IV) every 3 weeks, docetaxel 75–100 mg/m^2^ (IV) every 3 weeks, nab-PTX 100–260 mg/m^2^ (IV) every 3 weeks, or Ixabepilone 40 mg/m^2^ iv every 3 weeks. Maximum 6 cycles.	Increase in ORR	[90]
CQ or HCQ + Carboplatin/ Gemcitabine	Phase I trial, refractory advanced solid tumors	CQ 50 mg/day or HCQ 100–150 mg/day (OR) on 7–21 days + CPT 5 AUC (IV) on day 1 + GEM 1000 mg/day (IV) on days 1 and 8 for 21 days, 4 cycles	PR SD PD Improved PFS and OS	[96]
HCQ + GEM	Pancreatic carcinoma	Preoperative GEM 1500 mg/m^2^ + HCQ for 31 days until surgery	↑OS and PFS Partial histopathological response ↓CA19-9 level	[101]
CQ + IMA	Chronic phase CML	IMA 400–800 mg + CQ 400–800 mg (OR) daily for 48 weeks	No significant effect	[107]
HCQ + Everolimus	Advanced renal cell carcinoma	Everolimus 10 mg for 1 week + HCQ 600 mg/twice daily for 35–28 days	Partial response and stable disease ↑PFS	[115]
HCQ + Temsirolimus	Melanoma, colorectal carcinoma, head and neck cancer, and breast cancer	TEM 25 mg (IV) + HCQ 200–1200 mg/day (OR) daily for 4–6 weeks	Stable disease	[116]
CQ + Carmustine + IR	Glioblastoma multiforme (GBM)	Carmustine 200 mg/L once every 6 weeks + CQ 150 mg daily from 1 day after surgery + radiotherapy 6000 Gy	Longer survival Tumor remission	[117]
CQ + Carmustine + IR	Glioblastoma multiforme (GBM)	Carmustine 200 mg/L + CQ 150 mg daily from 5 day after surgery for 12 months + 6000 Gy, 4 cycles	Improved mid-term survival	[118]
				
HCQ + Bortezomib	Relapsed/refractory myeloma	2-week HCQ 100–1200 mg (OR) + Bortezomib 1–1.3 mg/m^2^ on days 1, 4, 8, and 11 of 21 d cycle	Partial response Minor response Stable disease	[120]
HCQ + CPT/PTX+/− Bevacizumab	Untreated metastatic non-small-cell lung cancer	PTX 200 mg/m^2^ (IV) on day 1 + CPT 6 AUC on day 1 +/− Bevacizumab 15 mg/kg (IV) on day 1 + CQ 200 mg (OR) on days 1–21 for 6 cycles	Modest improvement in RR ↑ORR and PFS in patients with Kras mutations	[122]
HCQ + GEM/nab-PTX	Pancreatic carcinoma	Two preoperative cycles of GEM 1000 mg/L + nab-PTX 125 mg/L on days 1, 8, and 15 + HCQ 1200 mg/day from day 1	Improved OS ↑Evans grade histopathologic tumor response ↑Tumor immune infiltration index	[123]
HCQ + GEM or HCQ + GEM + nab-PTX	Pancreatic carcinoma	1 month of preoperative GEM + HCQ 1200 mg/day or 2 months of GEM/nab-PTX + HCQ 600 mg twice daily	↑Evans grade histopathological responses in patients with SMAD4 loss. Improvement of biochemical markers	[124]
HCQ + GEM/nab-PTX	Metastatic pancreatic cancer	HCQ 600 mg/twice daily (OR) for 28 days + standard chemotherapy	No improvement of OS Partial response	[125]

Abbreviations: OS—overall survival, ORR—objective response rate, PFS—progression-free survival (PFS). ↑—increased expression, ↓—downregulation.

## Data Availability

Not applicable.
